# Rheumatoid synovial fluid interleukin-17-producing CD4 T cells have abundant tumor necrosis factor-alpha co-expression, but little interleukin-22 and interleukin-23R expression

**DOI:** 10.1186/ar3152

**Published:** 2010-10-07

**Authors:** Leigh D Church, Andrew D Filer, Esther Hidalgo, Katherine A Howlett, Andrew MC Thomas, Stephen Rapecki, Dagmar Scheel-Toellner, Christopher D Buckley, Karim Raza

**Affiliations:** 1 Rheumatology Research Group, MRC Centre for Immune Regulation, Institute for Biomedical Research, School of Immunity and Infection, College of Medical and Dental Sciences, University of Birmingham, Edgbaston, Birmingham, B15 2TT, UK; 2 Sandwell and West Birmingham Hospitals NHS Trust, Dudley Road, Birmingham, B18 7QH, UK; 3 The Royal Orthopaedic Hospital, Bristol Road South, Northfield, Birmingham, B31 2AP, UK; 4 UCB, 208 Bath Road, Slough, Berkshire, SL1 3WE, UK

## Abstract

**Introduction:**

Th17 cells have been implicated in the pathogenesis of rheumatoid arthritis (RA). The aim of this study was to systematically analyse the phenotype, cytokine profile and frequency of interleukin-17 (IL-17) producing CD4-positive T cells in mononuclear cells isolated from peripheral blood, synovial fluid and synovial tissue of RA patients with established disease, and to correlate cell frequencies with disease activity.

**Methods:**

Flow cytometry was used to analyse the phenotype and cytokine production of mononuclear cells isolated from peripheral blood (PBMC) (*n *= 44), synovial fluid (SFMC) (*n *= 14) and synovium (SVMC) (*n *= 10) of RA patients and PBMC of healthy controls (*n *= 13).

**Results:**

The frequency of IL-17-producing CD4 T cells was elevated in RA SFMC compared with RA PBMC (*P *= 0.04). However, the frequency of this population in RA SVMC was comparable to that in paired RA PBMC. The percentage of IL-17-producing CD4 T cells coexpressing tumor necrosis factor alpha (TNFα) was significantly increased in SFMC (*P *= 0.0068). The frequency of IFNγ-producing CD4 T cells was also significantly higher in SFMC than paired PBMC (*P *= 0.042). The majority of IL-17-producing CD4 T cells coexpressed IFNγ. IL-17-producing CD4 T cells in RA PBMC and SFMC exhibited very little IL-22 or IL-23R coexpression.

**Conclusions:**

These findings demonstrate a modest enrichment of IL-17-producing CD4 T cells in RA SFMC compared to PBMC. Th17 cells in SFMC produce more TNFα than their PBMC counterparts, but are not a significant source of IL-22 and do not express IL-23R. However, the percentage of CD4 T cells which produce IL-17 in the rheumatoid joint is low, suggesting that other cells may be alternative sources of IL-17 within the joints of RA patients.

## Introduction

Rheumatoid arthritis (RA) is a systemic chronic inflammatory disorder associated with persistent and destructive synovitis leading to cartilage and bone erosion. The underlying cause of RA is unknown; however, the pathogenesis of RA is thought to be the result of complex cell to cell interactions between amongst others, T cells, macrophages and fibroblasts. In established disease, the preponderance of IFNγ-expressing and paucity of IL-4-expressing T cells, *in situ *and *ex vivo*, had until recently led to the description of RA as an immune mediated inflammatory disease associated with a predominantly T helper type-1 (Th1)-like cytokine profile [1-3].

More recently, effector T cells (Th17 cells), that produce interleukin-17A (IL-17) [4,5] and that are functionally distinct from Th1 and Th2 helper T cells, have been identified in mice and subsequently in humans [6]. Th17 cells have an important role in the clearance of extracellular bacteria and fungi, but also appear to play a pathogenic role in several inflammatory and autoimmune diseases. In experimental animal models, IL-17-producing T cells are involved in the pathogenesis of experimental autoimmune encephalomyelitis (EAE), collagen-induced arthritis (CIA), colitis and psoriasis [7-9].

In mice, the development of Th17 cells is driven by the transcription factor retinoic acid-related orphan receptor γt (RORγt). Differentiation from naïve T cells requires TGFβ, IL-1, and IL-6 [10,11]. In humans, the origin of Th17 cells and the factors that regulate their development remain controversial, but like murine Th17 cells, IL-1 and IL-6 are essential and it is likely that TGF-β also plays a role. Both murine and human Th17 cells require IL-23 for their expansion and survival. Th17 differentiation is not only regulated by cytokines but also by environmental and dietary factors, such as aryl hydrocarbons [12,13] and vitamin D_3 _[14,15]. In addition to IL-17, Th17 cells have been shown to produce IL-21, IL-22, TNFα and IFNγ [16].

In RA, IL-17 has been detected in synovial fluid (SF) and synovium [17-21]. Its expression is associated with inflammation and joint destruction, as well as with production of IL-1β and TNFα. In addition to stimulating the production of these proinflammatory cytokines, IL-17 acts synergistically by amplifying their effects [22,23]. We have previously identified IL-17-producing T cells within SF and synovial tissue, and demonstrated that RA synovial fibroblasts treated with IL-17 and TNFα promote the survival and functional lifespan of neutrophils, contributing to the increased number of neutrophils observed in the rheumatoid synovial microenvironment [23]. Based upon the combined evidence for a role of IL-17 in inflammation, targeting of IL-17 is now being tested as a new therapeutic strategy for the treatment of RA [24].

However, relatively little is known about the phenotype, cytokine profile and frequency of Th17 cells in the synovial environment and how they relate to RA disease activity. Two reports have shown that the frequency of Th17 cells was increased in the blood of RA patients compared with healthy donors [15,25], whilst Shahrara *et al. *demonstrated that the percentage of Th17 cells was higher in RA SF compared with normal and RA peripheral blood [15,25,26]. In addition, IL-17-producing T cells were shown to be enriched in the joints of children with juvenile idiopathic arthritis [27]. In contrast, Yamada *et al. *reported fewer Th17 cells in the joints of RA patients compared to peripheral blood [28]. In light of these conflicting data, we investigated the phenotype and frequency of IL-17-producing T cells in the blood, SF and synovial tissue of patients with RA, examining the cytokine profile of this population and correlation with disease activity.

## Materials and methods

### Patients

Paired blood and SF was obtained from 14 RA patients (nine female) meeting the 1987 ACR criteria [29] with a median age of 60.5 years (interquartile range (IQR) 52 to 69.5), and a median DAS28 of 5.55 (IQR 4.41 to 6.11). Paired blood and synovium was obtained from 10 RA patients (nine female) meeting the 1987 ACR criteria [29] with a median age of 56 years (IQR 48 to 71), and a median DAS28 of 4.75 (IQR 4.08 to 5.95) undergoing joint replacement surgery. The clinical details of patients who donated blood and SF or synovium are given in Table 1. Peripheral blood samples were obtained from an additional 20 RA patients (10 female) giving a total of 44 RA blood samples analysed. Blood from 13 healthy control (HC) donors (six female) with a median age of 56 years (IQR 51.5 to 62) was obtained. Details of the 44 RA patients for whom analysis of blood was conducted are given in Table 2. Ethical approval for this study was given by the local research ethics committee and all subjects gave written informed consent.

**Table 1:** Characteristics of RA patients for whom analysis of IL-17-positive CD4 T cells in paired blood and synovial fluid (top), and paired blood and synovium (bottom) was performed.

				Treatment						% CD3+CD4+ IL17+	
Subject	RF	ACPA	Disease duration, years	DMARD	Anti-TNF	Steroid	**ESR**, mm/hr	**CRP**, mg/l	DAS28 (ESR)	PBMC	SFMC
1	+ve	+ve	<1	MTX, HCQ	N	N	39	15	6.20	1.95	2.52
2	+ve	+ve	2	MTX, HCQ	N	N	61	8	7.01	1.09	0.82
3	+ve	+ve	1	N	N	N	21	7	4.18	2.36	2.17
4	+ve	-ve	23	MTX	N	N	18	11	5.83	0.73	0.79
5	+ve	-ve	45	MTX	N	N	60	11	5.27	0.63	0.53
6	+ve	+ve	5	MTX, SSZ	N	N	69	93	5.48	2.34	2.84
7	+ve	+ve	4	N	Y	Y	28	30	3.51	3.48	1.17
8	+ve	+ve	<1	MTX, HCQ	N	Y	63	72	6.14	1.59	1.44
9	+ve	+ve	5	N	Y	Y	32	12	5.62	1.30	7.90
10	+ve	+ve	<1	MTX	Y	N	80	71	6.08	0.73	5.18
11	-ve	+ve	1	N	N	N	10	15	5.76	0.86	3.48
12	+ve	+ve	5	MTX	Y	Y	24	24	4.42	1.06	1.06
13	-ve	-ve	6	HCQ	N	N	81	92	4.39	0.37	4.45
14	+ve	+ve	1	MTX	N	Y	17	16	4.48	0.49	0.46
				**Treatment**						**% CD3+CD4+** **IL17+**	
**Subject**	**RF**	**ACPA**	**Disease duration, years**	**DMARD**	**Anti-TNF**	**Steroid**	**ESR**, **mm/hr**	**CRP**, **mg/l**	**DAS28** **(ESR)**	**PBMC**	**SVMC**
1	+ve	+ve	16	N	Y	N	20	20	3.83	0.77	0.55
2	+ve	+ve	4	N	Y	Y	13	9	4.95	0.57	0.48
3	+ve	+ve	5	N	Y	Y	13	3	4.50	1.63	0.78
4	-ve	+ve	8	MTX	Y	N	12	0	3.57	1.49	1.08
5	+ve	+ve	20	N	N	Y	89	24	6.03	2.63	0.91
6	+ve	+ve	23	N	N	Y	45	57	5.86	0.08	0.24
7	+ve	+ve	20	SSZ	N	N	31	11	4.33	0.73	0.81
8	+ve	+ve	5	N	Y	Y	15	3	4.55	0.29	1.17
9	-ve	-ve	15	N	Y	N	115	22	6.19	1.37	0.99
10	-ve	+ve	1	LEF	N	N	10	15	5.76	1.65	0.48

-ve, negative; +ve, positive; ACPA, anti-CCP antibodies; HCQ, hydroxychloroquine; LEF, leflunomide; MTX, methotrexate; N, no; PB, peripheral blood; RF, rheumatoid factor; SF, synovial fluid; SSZ, sulfasalazine; SVMC, synovium mononuclear cells; Y, yes.

**Table 2:** Summary of RA patients for whom analysis IL-17-positive CD4 T cells in the blood was conducted

RA patients (n)	44
Age, years (median (IQR))	56 (50 to 65.5)
Sex, female (n)	28
Disease duration, years (median (IQR))	3.5 (1.0 to 5.5)
RF positive (n)	32
ACPA positive (n)	30
ESR, mm/hr (median (IQR))	24 (10 to 40)
CRP, mg/ml (median (IQR))	11 (<5 to 23)
DAS28 (ESR) (median (IQR))	5.15 (4.01 to 6.17)
% IL17-positive CD4 T cells (median (IQR))	1.08 (0.60 to 1.64)
% IL17-positive CD45RO CD4 T cells (median (IQR))	1.10 (0.47 to 1.79)

IQR, interquartile range.

### Peripheral blood and synovial fluid cell preparation

Peripheral blood mononuclear cells (PBMC) and SF mononuclear cells (SFMC) were isolated by density gradient centrifugation on Ficoll-paque™-Plus (GE Healthcare, Amersham, UK), washed twice with RPMI-1640 medium (Sigma-Aldrich, St Louis, MO, USA), counted using a Neubauer hemocytometer and resuspended at 1 × 10^6 ^cells/ml in fresh medium.

### Synovial tissue dissociation

RA synovium was cut into small pieces and washed in RPMI-1640 medium. The tissue suspension was transferred into a Stomacher^® ^400 Circulator Bag (Seward Ltd, Worthing, UK), heat sealed, placed in a Stomacher^® ^400 circulator (Seward Ltd) and run at 230 rpm for five minutes. The contents of the bag were removed and passed through a BD Falcon 70 mm nylon cell strainer. The filtered cell suspension was then layered onto ficoll and synovial tissue mononuclear cells (SVMC) isolated from the buffy coat following centrifugation as above.

### Flow cytometric analysis

Monoclonal antibodies (mAb) and reagents used for flow cytometric analysis were phycoerythrin (PE)-conjugated anti-IL-17A mAb (eBioscience, San Diego, CA, USA), anti-IL-4 mAb (BD Biosciences, Franklin Lakes, NJ, USA), anti-CD4 mAb (Immunotools, Friesoythe, Germany); fluorescein isocyanate (FITC)-conjugated anti-interferon-γ mAb (Invitrogen, Paisley, UK), anti-IL-6 mAb (eBioscience), anti-TNFα mAb (BD Biosciences), anti-IL-10 mAb (eBioscience), anti-CD4 mAb (Immunotools), anti-CD45RO mAb (Dako, Fort Collins, CO, USA), anti-IL-23R mAb (R&D Systems, Abingdon, UK); allophycoerythrin (APC)-conjugated anti-IL-22 mAb (R&D Systems), anti-CD45RA mAb (Southern Biotech, Birmingham, AL, USA), anti-CD8 mAb (Biolegend, San Diego, CA, USA); pacific blue (PcB)-conjugated anti-CD4 mAb (eBioscience); PE-Cy7-conjugated anti-CD3 mAb (eBioscience); PE-Cy647-conjugated anti-CD14 mAb (Immunotools). Stained cells were run on a Cyan flow cytometer (Dako), and the data analysed using Summit v4.3 software (Dako).

### Intracellular staining of cytokines

Mononuclear cells were stimulated with 100 ng/ml of phorbol myristate acetate (PMA) (Sigma-Aldrich) and 1 μg/ml of ionomycin (Sigma-Aldrich) for three hours together with 2 μg/ml of brefeldin A (Sigma-Aldrich). This time-point was identified prior to this study, in a time-course experiment, as the optimum for intracellular cytokine measurement of IL-17 and IFNγ (the prime discriminators between Th1/Th17 cells) (data not shown). After surface staining, intracellular staining was performed using a Caltag fixation and permeabilisation kit (Invitrogen) according to manufacturer's instructions.

### Statistical analysis

Correlations were examined using Spearman rank test. For comparisons of unpaired and paired samples, the Mann-Whitney U test and Wilcoxon signed rank test were used respectively, with two-tailed *P-*values. Medians and interquartile ranges (IQR) are reported. *P-*values less than 0.05 were considered significant.

## Results

### Frequencies of IL-17-producing CD4 T cells in PBMC, SFMC and SVMC of patients with RA

**Figure 1:**
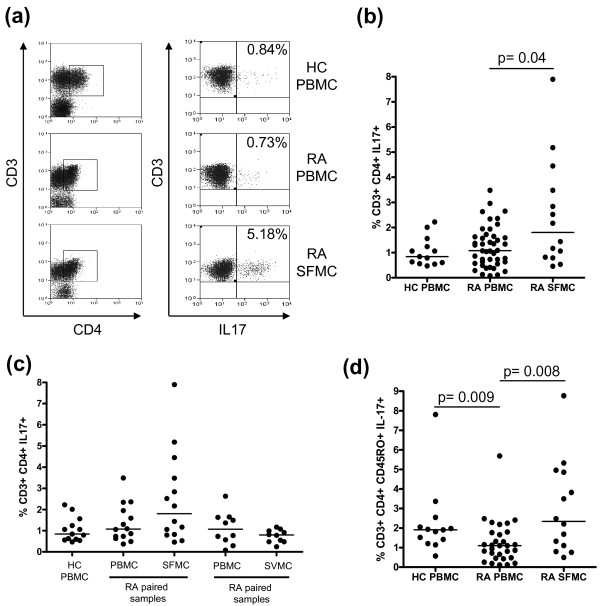
**Frequency of IL-17-positive CD4 T cells in PBMC, SFMC, and synovium (SVMC) of RA patients versus PBMC of healthy donors**. Cells were stimulated with PMA and ionomycin in the presence of brefeldin A. **A) **Representative plots of IL-17 staining on PBMC of healthy controls (HC), RA PBMC, and RA SFMC are shown against CD3. Cells were first gated on CD3 and CD4 expression. The percentages of IL-17 positive cells are indicated. **B) **The frequency of IL-17-positive CD4 T cells was compared between RA and healthy control samples. Data from healthy control PBMC (*n *= 13), RA PBMC (*n *= 44) and SFMC samples (*n *= 14) are shown. **C) **Data from healthy control PBMC (*n *= 13), RA paired PBMC and SFMC samples (*n *= 14) and paired RA PBMC and SVMC samples are shown (*n *= 10). **D) **The frequency of IL-17-positive CD4 CD45RO T cells was compared between healthy donor PBMC (*n *= 13), RA PBMC (*n *= 44) and RA SFMC (*n *= 14). Bars representing the medians are shown within the dot plots. *P-*values less than 0.05 were considered significant.

Flow cytometry was used to measure the intracellular expression of IL-17 following stimulation with PMA and ionomycin in CD4 T cells from peripheral blood, SF and mechanically dissociated synovial tissue of RA patients and from the peripheral blood of healthy donors. The frequencies of IL-17-producing CD4 T cells in PBMC and SFMC of patients with RA were variable. The median proportion of IL-17-producing CD4 T cells in SFMC (*n *= 14) (1.81% (0.81 to 3.97)) was significantly (*P *= 0.04) greater than in PBMC (*n *= 44) (1.08% (0.60 to 1.64)) from RA patients, and increased compared with PBMC (*n *= 13) (0.84% (0.58 to 1.40)) from healthy controls (Figure 1A, 1B). Examination of the frequency of IL-17-producing CD4 T cells in paired samples from RA patients also confirmed an enrichment of this population in the SFMC (1.81% (0.81 to 3.97)) compared with PBMC (1.08% (0.68 to 2.15)) (Figure 1C). Conversely, we observed a lower median proportion of IL-17-producing CD4 T cells in cells isolated from RA synovium (SVMC) (0.80% (0.48 to 1.04)) compared with PBMC (1.07% (0.43 to 1.64)) from those individuals (Figure 1C). However, none of these differences reached statistical significance.

As previous reports have identified the vast majority of IL-17-positive cells to be found within the memory (CD45RO) CD4 population, we examined the frequencies of IL-17-producing CD4-positive CD45RO-positive T cells from the peripheral blood and SF of RA patients and compared these with the peripheral blood of healthy donors (Figure 1D). The frequency of these cells amongst SFMC (*n *= 14) (2.35% (0.95 to 4.91)) was significantly higher (*P *= 0.008) compared to RA PBMC (*n *= 44) (1.10% (0.47 to 1.79)). The frequency of IL-17-producing CD4-positive CD45RO-positive T cells in PBMC of RA patients was significantly lower (*P *= 0.009) than the PBMC of healthy controls (*n *= 13) (1.91% (1.32 to 2.30)).

There were no significant correlations between the DAS28 or DAS28 components (CRP and swollen and tender joint counts) and the frequencies of IL-17-producing CD4 T cells in PBMC, SFMC, or SVMC in patients with RA. As only 3 of the 14 RA patients for whom we had obtained SF were anti-CCP antibody negative (ACPA -ve), we were unable to draw conclusions regarding the frequencies of IL-17-producing CD4 T cells in SFMC of anti-CCP antibody positive (ACPA +ve) *versus *ACPA -ve patients. However, there was no difference in the frequency of IL-17-producing CD4 T cells in the peripheral blood between ACPA +ve (1.00% (0.36 to 1.72)) and ACPA -ve (0.97% (0.38 to 1.94)) RA patients. Also, there were no correlations between the frequencies of IL-17-producing CD4 T cells and the DAS28, when ACPA +ve and ACPA -ve RA patients were examined separately. Examination of the DAS28 in patients segregated on the basis of a high frequency (≥1.0%) versus low frequency (<1.0%) of SF IL-17-positive CD4 T cells found no significant difference between these groups.

### IFNγ co-expression by IL-17-positive CD4 T cells in PBMC, SFMC and SVMC of patients with RA

**Figure 2:**
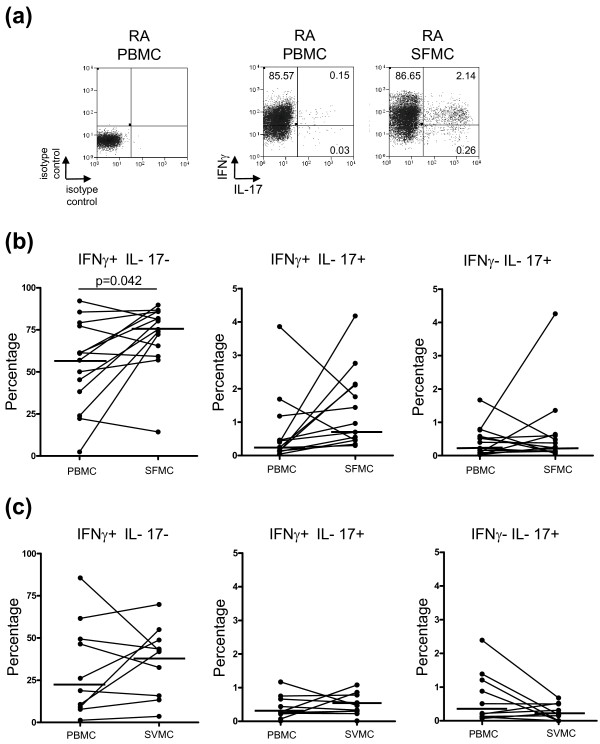
**IFNγ co-expression by IL-17-positive CD4 T cells in PBMC, SFMC, and SVMC of patients with RA**. **A) **Representative plots of the expression of IFNg against IL-17 in CD4 T cells from isolated PBMC and SFMC are shown. The frequencies of IFNγ-positive IL-17-negative, IFNγ-positive IL-17-positive, IFNγ-negative IL-17-positive cells gated on CD4 T cells in **B) **RA PBMC and SFMC, **C) **RA PBMC and SVMC, are shown. Bars representing the medians are shown within the plots.

RA has previously been deemed a Th1 disease and IL-17-producing CD4 T cells are capable of coexpressing the archetypical Th1 cytokine, IFNγ. We thus examined the frequency of IFNγ-positive CD4 T cells in relation to IL-17-positive CD4 T cells within blood, SF and SVMC of patients with RA. In both the peripheral blood and SF of RA patients, the majority of CD4 T cells expressed IFNγ (Figure 2A, 2B) with the frequency of IFNγ-positive IL-17-negative T cells significantly higher in the SFMC (77.75% (62.37 to 85.93)) compared to PBMC (59.97% (31.07 to 78.27)) (*P *= 0.042). There was no significant difference between the median frequency of IFNγ IL-17 double positive CD4 T cells amongst SFMC (0.83% (0.46 to 2.12)) and PBMC (0.31% (0.14 to 0.82)) or in the median frequency of the IFNγ-negative IL-17-positive subset between the PBMC (0.37% (0.12 to 1.30)) and SFMC (0.25% (0.12 to 0.62)). There was an inverse relationship between the frequencies of IFNγ-positive IL-17-negative cells and IFNγ-negative IL-17-positive CD4 T cells in SFMC (r = -0.66, *P *= 0.01).

The pattern of IFNγ and IL-17 expression in PBMC and SVMC CD4 T cells (Figure 2C), was similar to that seen in PBMC and SFMC (Figure 2B). There were no significant correlations between the frequencies of any of the subsets and the DAS28. In a previous study, the use of methotrexate (MTX) or prednisolone was associated with a higher frequency of IL-17-positive CD4 T cells in PBMC [28]. In our study however, we observed that the frequency of IL-17-positive CD4 T cells in PBMC (1.07% (0.59 to 1.77), *n *= 22) in patients taking MTX or prednisolone was marginally lower compared to patients not receiving these drugs (1.34% (0.53 to 1.64), *n *= 22). There was no difference in disease activity between these two groups. Of the 14 patients in whom SFMC were analysed, nine were taking MTX and these patients also had a lower frequency of IL-17-positive CD4 T cells in the SFMC (1.06% (0.66 to 2.68)) compared with the five patients not receiving MTX (3.48% (1.67 to 6.18)). Only one patient from whom synovial tissue was obtained was taking MTX, and therefore this association was not examined. There were also no differences between the frequencies of IL-17-positive CD4 T cells in patients receiving or not receiving biologic agents in PBMC, SFMC or SVMC.

### Analysis of IL-22 and IL-23R expression on IL-17-positive CD4 T cells in PBMC and SFMC of patients with RA

**Figure 3:**
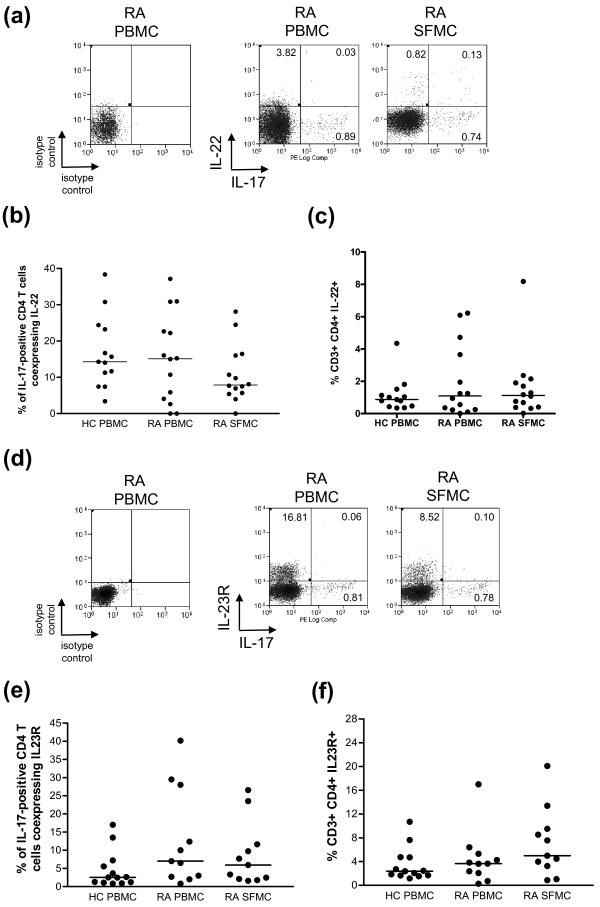
**Analysis of IL-22 and IL-23R expression on IL-17-positive CD4 T cells in PBMC and SFMC of patients with RA**. **A**) Representative plots of the expression of IL-22 against IL-17 in CD4 T cells from isolated PBMC and SFMC are shown. **B**) The percentages of IL-17-positive CD4 T cells coexpressing IL-22 in PBMC from healthy donors (*n *= 13), paired RA PBMC and SFMC samples (*n *= 14) are shown. **C**) The frequencies of IL-22-positive CD4 T cells in HC PBMC, RA PBMC and paired SFMC are shown. **D**) Representative plots of the expression of IL-23R against IL-17 in CD4 T cells from isolated PBMC and SFMC are shown. **E**) The percentages of IL-17-positive CD4 T cells coexpressing IL-23R in PBMC from healthy donors (*n *= 13) and paired RA PBMC and SFMC samples (*n *= 11) are shown. **F**) The frequency of IL-23R-positive CD4 T cells was compared between healthy donor PBMC (*n *= 13), RA PBMC and RA SFMC (*n *= 11). Bars representing the median are shown within the dot plots.

IL-22 is produced by Th17 cells [9,30,31] and has been implicated in RA [32,33], whilst IL-23 is thought to be critical for the expansion and survival of Th17 cells. We examined the frequency of IL-22 and IL-23-receptor (IL-23R) coexpression in IL-17-positive T cells within the SF and peripheral blood of patients with RA. IL-22 and IL-17 double positive CD4 T cells (Figure 3A) were hardly detectable in PBMC (0.07% (0.02 to 0.30)), or SFMC (0.16% (0.04 to 0.35)) of RA patients and PBMC (0.16% (0.07 to 0.21)) of healthy controls. The median percentage of IL-17-positive CD4 T cells coexpressing IL-22 did not significantly differ between SFMC (7.90% (5.57 to 16.23)), RA PBMC (15.13% (3.36 to 26.76)) and PBMC of healthy donors (14.29% (9.37 to 23.85)) (Figure 3B). Furthermore, the total frequency of IL-22-positive CD4 T cells was not significantly elevated in RA PBMC (1.09% (0.24 to 4.19)) and SFMC (1.12% (0.40 to 2.03)) compared with PBMC of healthy controls (0.87% (0.45 to 1.32)) (Figure 3C).

Despite the low frequency of IL-17-positive CD4 T cells coexpressing IL-22, the frequency of IL-22-positive CD4 T cells correlated strongly with the frequency of IL-17-producing CD4 T cells in PBMC of RA patients (r = 0.57, *P *< 0.0004); this correlation was not evident in SFMC (r = 0.35, *P *= 0.22). The frequency of IL-22-positive CD4 T cells in RA SFMC showed an inverse correlation with the DAS28 (r = -0.67, *P *= 0.008).

Interestingly, we detected very little expression of IL-23R on IL-17-positive CD4 T cells (Figure 3D, 3E). There was a non-significant trend towards a higher frequency of total CD4 T cells expressing IL-23R in RA SFMC (6.28% (3.63 to 9.07)) and RA PBMC (3.65% (2.07 to 5.31)) compared with the PBMC of healthy controls (2.38% (1.68 to 4.74)) (Figure 3F). In contrast to the IL-22-positive CD4 T cells, the frequency of IL-23R-positive CD4 T cells did not correlate with IL-17-producing CD4 T cells in either PBMC or SFMC compartments, nor was there any correlation with disease activity.

### Coexpression of TNFα by IL-17-producing CD4 T cells in PBMC and SFMC of patients with RA

**Figure 4:**
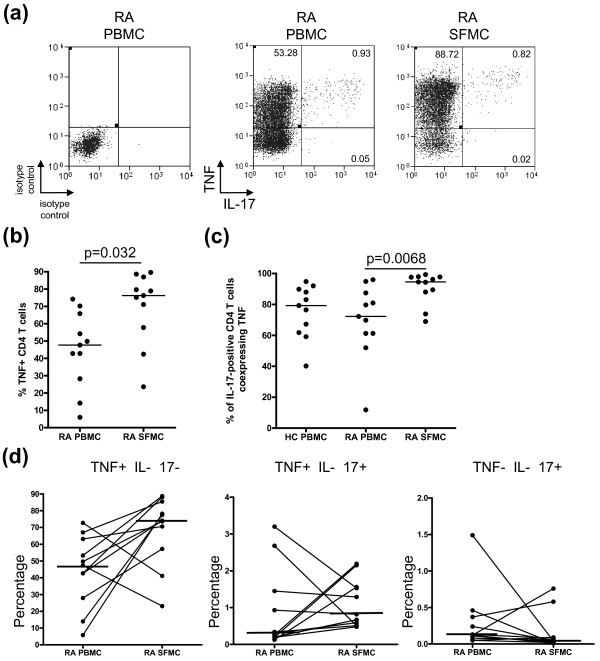
**Coexpression of TNFα by IL-17-producing CD4 T cells in PBMC and SFMC of patients with RA**. **A**) Representative plots of the expression of TNFα against IL-17 in CD4 T cells from isolated PBMC and SFMC are shown. **B**) The percentages TNFα-positive CD4 T cells in PBMC (*n *= 11) and SFMC (*n *= 11) of RA patients. **C**) The percentages of IL-17-positive CD4 T cells coexpressing TNFα in PBMC from healthy donors (*n *= 11), paired RA PBMC and SFMC samples (*n *= 11) are shown. **D**) The frequencies of TNFα-positive IL-17-negative, TNFα-positive IL-17-positive, TNFα-negative IL-17-positive gated on CD4 T cells in RA PBMC and SFMC are shown. Bars representing the median are shown within the dot plot. The results of statistical analysis are shown within the plots. P-values less than 0.05 were considered significant.

Given that TNFα plays an important role in the pathogenesis and persistence of RA, we examined whether IL-17-producing CD4 T cells coexpressed TNFα (Figure 4A). As anticipated, the frequency of TNFα-producing CD4 T cells in SFMC was significantly elevated (76.24% (57.77 to 86.97)) compared with PBMC (47.66% (28.24 to 65.79)) in patients with RA (*P *= 0.032) (Figure 4B). To determine whether IL-17-producing CD4 T cells were found within this population we determined the percentage of IL-17-positive CD4 T cells that coexpressed TNFα (Figure 4C). Despite almost all IL-17-positive CD4 T cells expressing TNFα this subset made up less than 1.0% of all CD4 T cells in the SFMC (0.82% (0.51 to 1.56)) compared with 74.09% (57.18 to 85.44) CD4 T cells expressing TNFα but not IL-17 in the SFMC (Figure 4D). However in RA SFMC, significantly more IL-17-positive CD4 T cells coexpressed TNFα (94.55% (88.06 to 97.77)) compared with only 72.22% (61.29 to 87.43) found in matched RA PBMC (*P *= 0.0068) and 79.27% (61.80 to 89.87) in healthy control PBMC.

Production of IL-6 by IL-17-producing CD4 T cells was also examined however most IL-17-producing CD4 T cells failed to produce IL-6 (data not shown).

## Discussion

There is considerable evidence, from both animal and human studies, that IL-17 plays a role in inflammatory arthritis [17-21,34]. Mechanistically, IL-17 promotes osteoclastogenesis, partly through upregulation of RANKL expression [35], and also synergises with other inflammatory cytokines, such as TNFα and IL-1, to amplify their effects [22,23]. These observations have led to the development of new therapeutic strategies aimed at targeting IL-17 or suppressing Th17 cells [24]. However, recent studies of the frequency of Th17 cells in RA patients have yielded conflicting data. Furthermore, the profile of cytokines produced by these cells *ex vivo *and their relationship with disease activity has not been well defined. Some studies have only examined this population in the blood of RA patients [25], whilst only two studies have examined these cells in SF or synovium [26,28].

We found no significant difference in the frequency of IL-17-producing CD4 T cells in the blood of RA patients and healthy controls. This contrasts with data from the study of Shen *et al. *(which looked at blood from 12 RA patients) but is consistent with data from Yamada *et al. *(which looked at blood from 123 RA patients and mononuclear cells isolated from the joints of 12 patients) [25,28]. The study by Shen *et al.*, whilst primarily focussed on Th17 cells in ankylosing spondylitis, demonstrated an increased frequency of IL-17-producing CD4 T cells in RA patients blood compared to healthy controls which correlated with both CRP and swollen joint counts (SJC). We would suggest that the larger sample sizes in our study (blood examined from 44 RA patients) and the study of Yamada *et al. *may provide a broader representation of what is recognised to be a very heterogeneous disease.

Our observation of a small but significant increase in the frequency of IL-17-producing CD4 T cells in SF compared to peripheral blood of RA patients, and the same for the frequency of memory CD4 T cells (CD45RO-positive) producing IL-17, are consistent with data from Shahrara *et al. *[26] but differ from the findings of Yamada *et al. *[28]. It should be noted however that Yamada *et al. *studied cells from the joints of only 12 RA patients and data from synovial fluid (*n *= 8) and synovial tissue (*n *= 4) were grouped together for analysis; this may be relevant in the context of our observation that the percentage of Th17 cells was lower in synovial tissue than in the blood of RA patients whereas the reverse was seen when synovial fluid was compared with blood.

Our data also demonstrate that there is marked variability in the frequency of IL-17-positive CD4 T cells in SF between RA patients. The underlying cause for this remains unclear but drug therapy may be a contributing factor. As discussed, Yamada *et al. *reported that patients taking prednisolone or MTX had a higher frequency of IL-17-producing CD4 T cells, but could not attribute this directly to treatment [28]. In our study we did not observe this phenomenon. Most patients in this study were receiving treatment with a non-steroidal anti-inflammatory drug, a disease modifying anti-rheumatic drug, a biologic agent or a combination of these (Table 1). We assessed the relationships between therapy and frequencies of IL-17-positive CD4 T cells but found no significant relationships. As it is not yet known how current therapeutic regimes affect the frequency of IL-17-producing CD4 T cells, it is possible that medication, in addition to other factors, such as disease duration and seropositivity, may contribute to the heterogeneity in frequency of IL-17-producing CD4 T cells amongst patients studied here. In the context of medication, appropriately designed longitudinal studies would be needed to address the impact of drug therapy on IL-17 producing cell populations.

IL-17-producing T cells were originally misclassified under the Th1 umbrella. Since the characterisation of Th17 cells, it has been identified that some IL-17-producing T cells coexpress IFNγ. This IFNγ-positive IL-17-positive subset is particularly enriched in the gut of patients with Crohn's disease [36]. Here we found that the majority of IL-17-producing CD4 T cells in the blood and SF of patients with RA also coexpress IFNγ. However, in the SF we observe a greater frequency of IFNγ-positive IL-17-negative CD4 T cells compared with blood, suggesting that Th1 cells and not Th17 cells predominate in the established RA joint. The relative paucity of IL-17-positive CD4 T cells in either the SF or synovium is at odds with the high levels of IL-17 detected in RA SF [17-21]. One explanation may lie with the use of a non-physiological stimulus *in vitro*, such as PMA/ionomycin; stimuli in the RA joint, such as antigen or cytokines, may drive a different cytokine response *in vivo*. Ziolkowska *et al. *demonstrated that RA PBMC produced significantly more IL-17 than SFMC when challenged with PMA/ionomycin *in vitro*, however, stimulation with IL-15 induced greater IL-17 production from SFMC than PBMC [21]. It is thus possible that the inflammatory environment of the rheumatoid synovium drives Th17 cells to produce IL-17 in a cytokine-dependent manner. In addition, the concept that CD4 T cells may not be the only source of IL-17 in the joint is being increasingly recognised. Mast cells have recently been identified as a source of IL-17 in RA synovium and are potent producers of IL-17 upon stimulation with TNFα, immune complexes and LPS [37].

IL-22 has been characterised as one of the effector cytokines secreted by Th17 cells [9,30,31], but it is also produced by Th1 cells [38]. Although this novel cytokine belongs to the IL-10 family, it is proinflammatory in function [39]. In animal models such as CIA, IL-22 is proinflammatory, whilst IL-22(-/-) mice are less susceptible to CIA than wild-type mice [32]. In human studies, IL-22 has been proposed to play a role in the pathogenesis of autoimmune inflammatory diseases, such as psoriasis [40] and Crohn's disease [41,42]. In RA, expression of IL-22 was found to be upregulated in synovium and capable of inducing synovial fibroblast proliferation and chemokine production [33]. Here, we found that IL-17-producing CD4 T cells in the SF coexpressed very little IL-22. Furthermore, we identified an IL-22-producing CD4 T cell population distinct from IL-17-producing CD4 T cells, but this population was not elevated in the blood of RA patients compared to healthy controls nor was it enriched in RA SF. This CD4 T cell subset may be similar to that recently reported to secrete IL-22 but not IL-17 and to be involved in the skin pathophysiology of psoriasis [43,44]. The paucity of IL-22-producing CD4 T cells in SF lends support to the notion that the primary source of IL-22 in the joint is not T cells but rather synovial fibroblasts and/or macrophages as reported by Ikeuchi *et al. *[33].

IL-22 production by Th17 cells has been shown to be dependent upon IL-23 [38,45,46], thus our observation, which corroborates that of Shen *et al. *[25], that very few IL-17-producing CD4 T cells express IL-23R provides a potential explanation for the paucity of IL-22-positive IL-17-positive CD4 T cells we observed. Considering the importance of IL-23 to the development and maintenance of Th17 cells, it is striking that over 90% and 85% of IL-17-producing CD4 T cells in the SF and blood respectively did not express IL-23R. However, our data are consistent with recent evidence showing that bioactive IL-23 (p19/p40) was barely detectable in joints of patients with RA [47].

In contrast to the pattern of IL-22 expression, the majority of IL-17-producing CD4 T cells in SF coexpressed TNFα; these double positive cells were significantly elevated in SF compared to blood. Although the percentage of IL-17-producing CD4 T cells coexpressing TNFα is very small compared to that of TNFα-producing CD4 T cells found in the SF, this may be relevant in terms of therapy. One of the mechanisms by which monoclonal antibodies against TNFα mediate their therapeutic effects, in addition to TNFα blockade, may be through induction of apoptosis in monocytes and T cells by outside to inside signalling through transmembrane TNFα [48-50]. Membrane expression of TNFα on IL-17-producing CD4 T cells would make these cells a potential target of monoclonal antibodies and may help to explain the beneficial effects of these therapies.

Upon identification of the IL-17/IL-23 axis, Th17 cells were viewed as a driving force in the pathogenesis of several autoimmune diseases previously associated with a Th1 predominance. In the case of multiple sclerosis evidence has supported this shift in paradigm [51,52]. However in RA, evidence is less clear. There is no doubt that cytokines associated with the Th17 lineage, such as IL-17, IL-6, IL-1β, IL-22, can be found within the RA joint [20,33], and as demonstrated here, there is evidence for a small but significant enrichment of Th17 cells within RA SF. In our study, however, we have only examined established RA and many patients had longstanding severe disease; thus 60% (6 of 10) of patients from whom synovial tissue was obtained and 29% (4 of 14) from whom synovial fluid was obtained were on anti-TNF agents. Recent data from Colin *et al. *and previous data from our group suggest that the situation may be different in the early phase of RA [15,20]. Further studies are required to define the role of Th17 cells in the early phase of disease. The absence of an enrichment of Th17 cells in the synovial tissue of established RA patients is paradoxical to the role this cytokine is reported to play in the pathogenesis of RA [17-21]. This leaves the possibilities that Th17 cells located outside the synovium, such as the juxta-articular bone marrow, may be an additional source of IL-17, that other cells in the synovium, including mast cells [37] may also produce IL-17 or that the experimental techniques used to reveal IL-17 production *ex vivo *do not reveal the true potential of T cells in the rheumatoid joint to produce IL-17. Future work will need to address these issues.

## Conclusions

Our findings demonstrate a modest enrichment of IL-17-producing CD4 T cells in RA synovial fluid compared to peripheral blood. Th17 cells in synovial fluid produce more TNF than their peripheral blood counterparts, but are not a significant source of IL-22 and do not express IL-23R. However, the percentage of CD4 T cells which produce IL-17 in the rheumatoid joint is low, suggesting that other cells may be alternative sources of IL-17 within the joints of RA patients.

## Abbreviations

+ve: positive; -ve: negative; ACPA: anti-CCP antibodies; CIA: collagen-induced arthritis; CRP: C-reactive protein; EAE: experimental autoimmune encephalomyelitis; ESR: erythrocyte sedimentation rate; FITC: fluorescein isocyanate; FSC: forward scatter; HCQL: hydroxychloroquine; IFN: interferon; IL: interleukin; IQR: interquartile range; LEF: leflunomide; mAb: monoclonal antibody; MTX: methotrexate; PB: peripheral blood; PBMC: peripheral blood mononuclear cells; PcB: pacific blue; PE: phycoerythrin; PMA: phorbolmyristate acetate; RA: rheumatoid arthritis; RF: rheumatoid factor; RORγt: retinoic acid-related orphan receptor γt; SF: synovial fluid; SFMC: synovial fluid mononuclear cells; SJC: swollen joint count; SSC: side scatter; SVMC: synovium mononuclear cells; SSZ: sulfasalazine; Th1: T helper type-1;TNF: tumor necrosis factor

## Competing interests

KR, DST and CDB hold an unrestricted research grant from UCB for work on the pathobiology of RA which supported the work described in this report. KR, ADF and CDB hold an unrestricted research grant from Cellzome for work on the pathobiology of RA. KR and CDB hold an unrestricted research grant from Pfizer for work on the pathobiology of RA. SR is employed by UCB.

## Authors' contributions

LDC conducted the majority of the experimental study, performed the statistical analysis and drafted the manuscript. AF was involved in the design of the study, consenting and collecting patient samples, coordinating patient samples with the Royal Orthopaedic Hospital, and helped with drafting the manuscript. EH and KH assisted in conducting the experimental study. AT was involved in the design of the study, consenting and collecting patient samples. SR was involved in the design and conception of the study. DST was involved in the design of the study and helped with drafting the manuscript. CB and KR were involved in the design and conception of the study, consenting and collecting patient samples and helped with drafting the manuscript. All authors read and approved the final manuscript.
